# A Rare Presentation of Sarcoidosis as a Pancreatic Head Mass

**DOI:** 10.1155/2017/7037162

**Published:** 2017-02-21

**Authors:** Shruti Mony, Pradnya D. Patil, Rebekah English, Ananya Das, Daniel A. Culver, Tanmay S. Panchabhai

**Affiliations:** ^1^Department of Internal Medicine, St. Joseph's Hospital and Medical Center, Phoenix, AZ, USA; ^2^Department of Hospital Medicine, Medicine Institute, Cleveland Clinic, Cleveland, OH, USA; ^3^Norton Thoracic Institute, St. Joseph's Hospital and Medical Center, Phoenix, AZ, USA; ^4^Arizona Center for Digestive Diseases and St. Joseph's Hospital and Medical Center, Phoenix, AZ, USA; ^5^Department of Pulmonary Medicine, Respiratory Institute, Cleveland Clinic, Cleveland, OH, USA

## Abstract

Sarcoidosis is a multisystem granulomatous syndrome of unknown etiology with noncaseating epithelioid granulomas being the pathognomonic pathological finding. Sarcoidosis most commonly involves the lungs and involvement of the gastrointestinal (GI) tract is uncommon. Pancreatic sarcoidosis is very rare, especially when it is the presenting feature of sarcoidosis and can masquerade as pancreatic cancer. Tissue infiltration in pancreatic sarcoidosis can lead to either a diffuse nodular appearance or a mass-like lesion. We present an interesting case of a 47-year-old woman with a 10-pack-year history of smoking who presented with sharp epigastric pain, weight loss, and elevated lipase level. CT and MRI imaging showed a 4 cm × 5 cm heterogeneous pancreatic mass with a dilated pancreatic duct and peripancreatic lymphadenopathy. Endoscopic ultrasound guided FNA revealed noncaseating granulomas with no evidence of malignancy or atypical infection. CT of the chest revealed bilateral mediastinal and hilar adenopathy with calcification, without any parenchymal abnormalities, and her angiotensin-converting enzyme level was elevated at 170 U/L. The clinical picture pointed to the diagnosis of pancreatic sarcoidosis. Given the severity of gastrointestinal symptoms related to pancreatic sarcoidosis, prednisone therapy at 0.5 mg/kg/day was initiated with complete resolution of symptoms at 8 weeks.

## 1. Introduction

Sarcoidosis is a multisystem granulomatous syndrome of unknown etiology. The pathognomonic histological finding is noncaseating epithelioid granulomas that contain multinucleated giant cells without an ascertainable cause (e.g., infection, foreign bodies). Sarcoidosis can involve virtually all organ systems to a varying extent and degree, but it usually affects the lungs (90%) and lymphoid system (30%). Common extrapulmonary organs involved in sarcoidosis are the liver, spleen, eyes, joints, and heart. Gastrointestinal involvement occurs in 0.1%–0.9% patients with sarcoidosis, most commonly affecting the liver and the stomach [1]. The gastrointestinal tract is typically involved secondary to organ infiltration or compression by enlarged lymph nodes, and most patients with gastrointestinal involvement are asymptomatic. Diagnosing intra-abdominal sarcoidosis is particularly challenging, as the lesions mimic neoplastic processes (e.g., lymphoma, carcinoma, or metastases) or necrotizing granulomatous infections (e.g., coccidioidomycosis).

## 2. Case Report

A 47-year-old white woman presented with sharp epigastric pain associated with flu-like symptoms of malaise, fatigue, and joint pain. Her symptoms had been waxing and waning in nature for 1-2 months but had worsened considerably in the week before presentation. She also complained of early satiety, loss of appetite, and weight loss of 30 pounds. She did not report recent travel or a history of fever, cough, or sick contacts. She was a native of Arizona and had a 10-pack-year smoking history. She denied alcohol use or intravenous drug use. Physical examination was notable for tachycardia and tenderness in the epigastric and periumbilical region without peritoneal signs.

**Figure 1:**
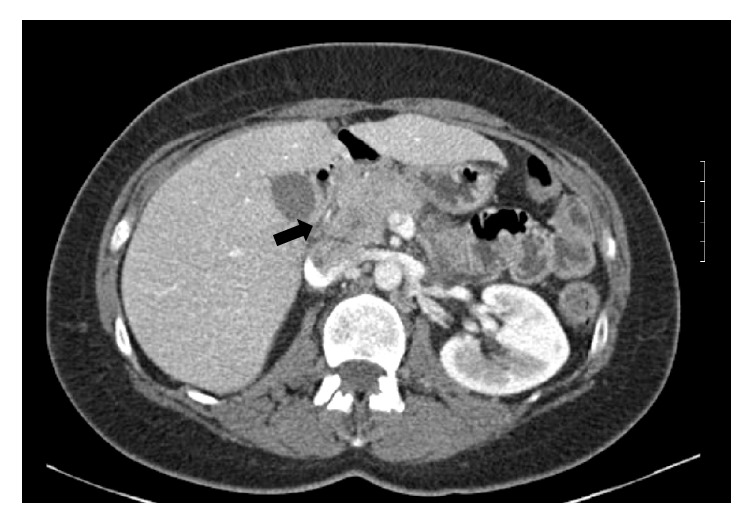
Computed tomogram of the abdomen and pelvis with contrast show abnormal fullness in the region of the pancreatic head measuring 4 × 1.5 cm (black arrow) with a dilated pancreatic duct measuring 5 mm.

**Figure 2:**
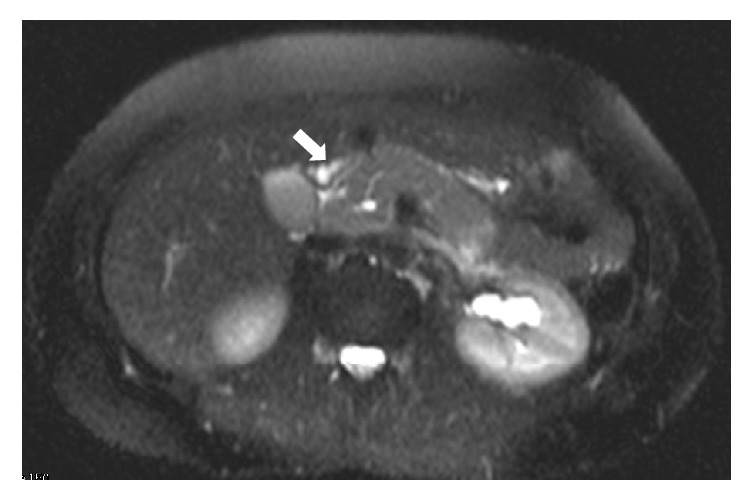
Magnetic resonance image of the abdomen with contrast (T2 sequence) shows heterogeneous signal pancreatic head mass measuring 4.5 cm, triggering concern for primary pancreatic carcinoma with associated pancreatic duct dilatation and peripancreatic lymphadenopathy (white arrow).

Laboratory data were significant for elevation in lipase of 428 U/L (Normal < 70 U/L). Her complete blood cell count and her liver and kidney function tests were normal. Abdominal computed tomogram (CT) with contrast showed an abnormal mass-like fullness of the pancreatic head (measuring 4 × 1.5 × 2 cm) with a dilated pancreatic duct (5 mm) but normal common bile duct (4 mm) (Figure 1). Magnetic resonance imaging (MRI) of the abdomen was carried out to visualize the biliary ducts; this imaging revealed a 4.5 × 5 cm heterogeneous pancreatic head mass, with pancreatic ductal dilation (1.4 cm) and peripancreatic lymphadenopathy concerning for primary pancreatic malignancy (Figure 2).

**Figure 3:**
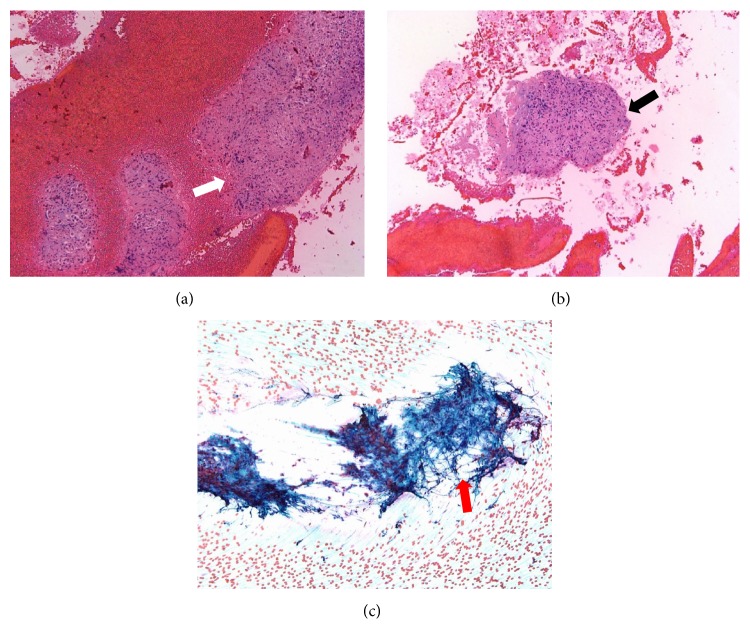
(a) Hematoxylin and eosin stain, 20x magnification, cell block, epithelioid, and noncaseating granuloma in a background of blood (white arrow). (b) Pancreatic mass fine needle aspiration, Papanicolaou stain, 20x magnification, and cell block, showing small fragments of epithelioid, noncaseating granuloma (black arrow). (c) Pancreatic mass fine needle aspiration, Papanicolaou stain, 40x magnification, smear showing epithelioid, and noncaseating granuloma (red arrow).

Due to the concern of malignancy, she underwent EUS-guided FNA. The pathology revealed noncaseating granulomas with no evidence of malignancy, cellular atypia, or infectious agents such as coccidioidomycosis (Figure 3). With nonnecrotizing granulomas seen on FNA, the patient underwent extensive work-up, including serology for coccidioidomycosis, cytomegalovirus, Epstein-Barr virus, and other autoimmune disorders. Colonoscopy showed no evidence of Crohn's disease. A CT of the chest revealed bilateral mediastinal and hilar adenopathy with calcification, without any parenchymal abnormalities. Pulmonary function testing was normal, and her angiotensin-converting enzyme level was elevated at 170 U/L. There was no evidence of cardiac involvement. During follow-up, the patient was diagnosed with erythema nodosum and sarcoid uveitis. Given the severity of gastrointestinal symptoms related to pancreatic sarcoidosis, prednisone therapy at 0.5 mg/kg/day was initiated and continued for 8 weeks. At 8 weeks, all of her gastrointestinal symptoms had resolved. Her prednisone was then tapered by 10 mg every 2 weeks. At 16 weeks, she was completely weaned off prednisone. CT abdomen-pelvis done 3 months after prednisone was completely weaned off showing complete resolution of the pancreatic mass lesion. Her sarcoid uveitis is now completely managed with topical corticosteroids.

## 3. Discussion

Pancreatic sarcoidosis with systemic features is rare and its initial presentation with primary pancreatic involvement associated with symptoms is even more uncommon [2]. It can masquerade as pancreatitis (acute or chronic) or pancreatic adenocarcinoma. Tissue infiltration in pancreatic sarcoidosis can either be diffusely nodular or a mass-like lesion of the pancreatic head. Associated symptoms most commonly include abdominal pain (50%), weight loss (44%), and obstructive jaundice (44%) [2]. Retroperitoneal or peripancreatic lymphadenopathy occurs in two-thirds of these patients. Acute symptoms related to pancreatic involvement seem to be more common in younger patients (18 to 47 years) commonly accompanied by hyperamylasemia and hypercalcemia. Older individuals with pancreatic involvement from sarcoidosis usually present with symptoms akin to chronic pancreatitis [3].

Because abdominal sarcoidosis is uncommon and is often asymptomatic, imaging plays a pivotal role in its diagnosis and management, but tissue diagnosis is ultimately required for definite diagnosis [4]. Imaging modalities such as ultrasound and CT show nonspecific features including an ill-defined pancreatic head mass, narrowing, and dilatation of the common bile duct with or without pancreatic duct dilatation, and enlarged lymph nodes [5]. Features on MRI imaging include low-signal intensity on T1-weighted images, mild-high signal intensity on T2-weighted images, and decreased enhancement compared with the normal pancreas after gadolinium administration [6]. Cases of high PET avidity in the pancreas in patients with sarcoidosis have been reported; however, no consensus currently exists on the best imaging modality for evaluation of pancreatic sarcoidosis [7]. Despite radiological advances, pancreatic masses are often difficult to diagnose until histological examination of biopsies or, in uncertain cases, operative exploration. In patients who have clinical evidence of sarcoidosis such as mediastinal adenopathy and peribronchovascular and perilymphatic lung nodules, the presence of a concurrent pancreatic mass lesion warrants tissue diagnosis to rule out malignancy. Pancreatic tissue sampling may be carried out via endoscopic ultrasound (EUS) or CT-guided fine needle aspiration (FNA). Anecdotal reports have suggested treatment with steroids, steroid-sparing immunosuppressive agents, or both [1, 4]. Factors that may require surgical intervention (e.g., a Whipple procedure) include large masses or complications such as massive hemorrhage, stricture, obstruction, or perforation [8]. Surgical intervention should be a last resort, only to be carried out when medical therapies have failed. Physicians must thus be cognizant of pancreatic sarcoidosis and its ability to masquerade malignancy [8, 9]. Due to the devastating nature of pancreatic adenocarcinoma, masses in the pancreas must be thoroughly investigated before confirming this diagnosis.

## References

[1] Bacal D., Hoshal V. L., Schaldenbrand J. D., Lampman R. M. (2000). Sarcoidosis of the pancreas: case report and review of the literature. *American Surgeon*.

[2] Warshauer D. M., Lee J. K. T. (2004). Imaging manifestations of abdominal sarcoidosis. *American Journal of Roentgenology*.

[3] Siavelis H. A., Herrmann M. E., Aranha G. V., Garcia G., Eubanks T., Reyes C. V. (1999). Sarcoidosis and the pancreas. *Surgery*.

[4] Sprague R., Harper P., McClain S., Trainer T., Beeken W. (1984). Disseminated gastrointestinal sarcoidosis. Case report and review of the literature. *Gastroenterology*.

[5] Mayne A. I., Ahmad J., Loughrey M., Taylor M. A. (2013). Sarcoidosis of the pancreas mimicking adenocarcinoma. *BMJ Case Reports*.

[6] Baroni R. H., Pedrosa I., Tavernaraki E., Goldsmith J., Rofsky N. M. (2004). Pancreatic sarcoidosis: MRI features. *Journal of Magnetic Resonance Imaging*.

[7] Okoro N., Moldovanyi C., Wehbi M., Obideen K. (2006). Sarcoidosis masquerading as pancreatic cancer. *Practical Gastroenterology*.

[8] Cook J., Spees T., Telefus P., Ranaudo J. M., Carryl S., Xiao P. (2013). Pancreatic sarcoidosis discovered during Whipple procedure. *Journal of Surgical Case Reports*.

[9] Yao Y., Jiang F., Jin Z. (2016). Pancreatic sarcoidosis in association with type 1 autoimmune pancreatitis: a case report and literature review. *Pancreas*.

